# Extramural depth of rectal cancer tumor invasion at thin-section MRI: predicting treatment response to neoadjuvant chemoradiation

**DOI:** 10.18632/oncotarget.4623

**Published:** 2015-08-06

**Authors:** Tong Tong, Yiqun Sun, Sanjun Cai, Zhen Zhang, Yajia Gu

**Affiliations:** ^1^ Department of Radiology, Fudan University Shanghai Cancer Center, Department of Oncology, Shanghai Medical College, Fudan University, Shanghai, P.R. China; ^2^ Department of Colorectal Surgery, Fudan University Shanghai Cancer Center, Department of Oncology, Shanghai Medical College, Fudan University, Shanghai, P.R. China; ^3^ Department of Radiotherapy, Fudan University Shanghai Cancer Center, Department of Oncology, Shanghai Medical College, Fudan University, Shanghai, P.R. China

**Keywords:** MRI, depth of invasion, rectal cancer, neoadjuvant therapy

## Abstract

**Objectives:**

To assess whether the maximal extramural depth (EMD) of T3 tumor spread on magnetic resonance imaging(MRI) correlates with tumor response parameters and whether it can predict tumor response to neoadjuvant chemoradiation.

**Methods:**

111 rectal cancer patients with American Joint Committee on Cancer (AJCC) T3 tumors underwent MRI staging before neoadjuvant chemoradiotherapy were included. Tumor EMD was measured as mm tumor beyond the muscular and compared between the following groups by Kruskal-Wallis test: pathological complete response(pCR) versus nonpCR, good regression versus poor regression, downstage versus nondownstage.

**Results:**

The final study population consisted of the 111 patients (79 male, 32 female). Median age was 56 years (range, 23–75 years). The EMD was significantly higher in nonpCR patients (7.8 ± 3.2 mm) than in pCR patients(6.1 ± 1.8 mm) (*p* = 0.033). According to good regression (tumor regression grade(TRG) 0–1 vs. TRG 2–3) and downstaging (ypStage 0-I vs. ypStage II–III), the difference was not significant. Receiver operating characteristic curve analysis revealed a good value for the area under the curve (0.775) and the cutoff value for EMD to predict pCR was 5.6 mm. Compared with patients with a EMD ≥ 5 mm, more patients with EMD <5 mm showed pCR (*p* = 0.019), while there was no correlation between EMD and good regression or downstaged.

**Conclusion:**

EMD value obtained on initial staging MRI may serve as an imaging biomarker which predicts patients that have an incomplete response pathological response after standard neoadjuvant therapy.

## INTRODUCTION

The current trends in the treatment of rectal cancer point toward a more widespread acceptance of neoadjuvant therapies. This creates an increasing need for preoperative imaging methods to noninvasively select high-risk patients who could benefit from the more aggressive multimodality treatment approaches [1]. Some authors reported a prognostic influence of the mesorectal fat infiltration depth and have suggested that this parameter should be included in therapeutic decision making [2–4). Depth of tumor invasion outside the muscularis propria has substantial clinical significance. The cancer-specific survival rate drops from 85% to 54%, independent of nodal involvement, when the depth of tumor invasion outside the muscularis propria exceeds 5 mm [5]. The definitions of stage T3a–T3c tumors have been taken from the standardized MR reporting criteria incorporated into the Radiological Society of North America's radiology reporting template for primary rectal cancer (T3a:tumor extends <5 mm beyond the muscularis propria; T3b:tumor extends 5–10 mm beyond the muscularis propria; T3c: tumor extends >10 mm beyond the muscularis propria) [6].

Current preoperative staging techniques include digital rectal examination [7], endorectal ultrasonography (US) [8], and computed tomography (CT) [9]. However, these modalities have not been shown to enable accurate measurement of the local depth of tumor spread. High-resolution MRI has become an important component of rectal cancer staging and multidisciplinary treatment planning, replacing other primary tumor-staging modalities in many centers. The results of the MERCURY Study demonstrate that MR imaging is feasible and reproducible in a multicenter setting and yields data equivalent to histopathologic results regarding the preoperative prediction of the depth of extramural tumor spread [10]. Another MERCURY Study also confirms the ability of MRI to select patients who are likely to have a good outcome with primary surgery alone [11].

The relevant parameters to evaluate tumor response include pCR, TNM downstaging and tumor regression grade (TRG). Several retrospective studies have found these parameters to be significant predictors of long-term outcomes such as local control and patient survival [12, 13]. If the depth of extramural tumor spread, as measured using thin-section MR, were to correlate with these parameters, predicting the prognosis of each patient would be facilitated. In addition, such data would be useful in identifying appropriate candidates for consideration of organ-preserving nonsurgical treatment strategies. Our previous study suggested that maximal extramural depth (EMD) value had the potential to become an imaging biomarker of tumor biological profile [14]. In this study, in this study we aim to assess whether the EMD of T3 tumor spread on MRI were correlated to tumor response parameters and whether it could predict tumor response to neoadjuvant chemoradiation.

## RESULTS

### Patient characteristics

A total of 111 patients were analyzed in the present study. The patient characteristics are summarized in Table 1. The study population was predominantly male (71.2%) and had a median age of 56 years (range, 23–75). 59 (53.2%) patients had tumor length less than 5 cm. The distance from the tumor to the anal verge was less than 5 cm in 46(41.4%) patients. The majority of tumors had 5 to 10 mm of penetration into the mesorectum (*n* = 79, 71.2%); however, 16.2% (*n* = 18) of the patients had >10 mm penetration. Lymph node metastases were assessed to be present in about three-forth of the patients (*n* = 81, 73.0%). 26 (23.4%) patients were assessed with MRI threatened circumferential resection margins. Interobserver agreement of confidence levels for observers 1 and 2 was good for EMD measurement (*k* = 0.632) and cN stage (*k* = 0.683) and was excellent for CRM evaluation (*k* = 0.861).

**Table 1:** Patient characteristics

characteristics	Value
Gender (*n*)	
Male	79 (71.2)
Female	32 (28.8)
Age (years [IQR])	56 (23–75)
Tumor length	
<5 cm	59 (53.2)
≥5 cm	52 (46.8)
Distance from the anal verge	
<5 cm	46 (41.4)
≥5 cm	65 (58.6)
mrTstage	
T3a	14 (12.6)
T3b	79 (71.2)
T3c	18 (16.2)
mrNstage	
N−	30 (27.0)
N+	81 (73.0)
CRM	
−(>1 mm)	85 (76.6)
+(≤1 mm)	26 (23.4)

Data presented as number of patients (*n* = 111), with percentages in parentheses.

### EMD according to postoperative pathologic findings

**Figure 1:**
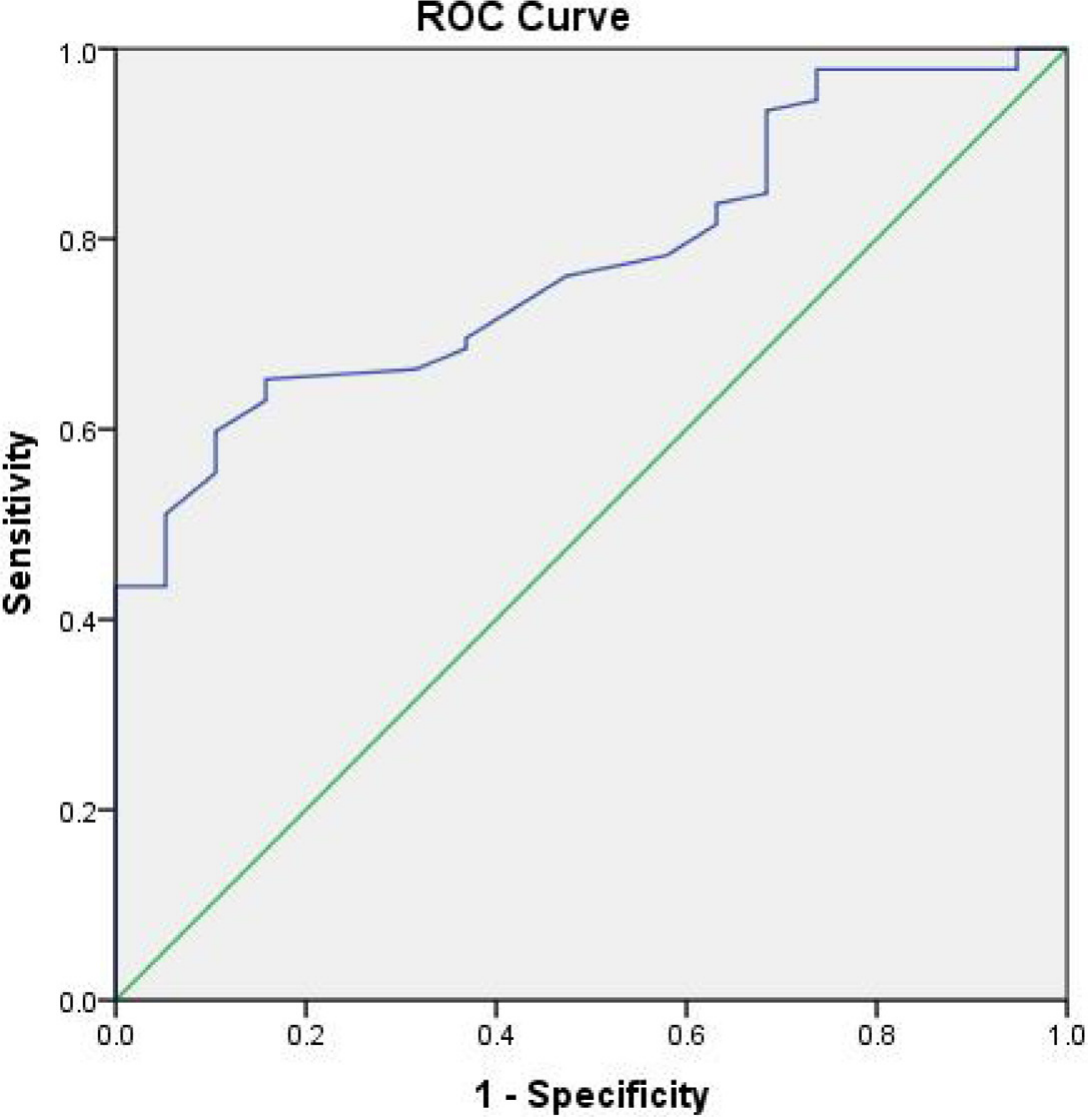
Receiver operator characteristics (ROC) curve for EMD before treatment (5.6 indicates best cut-off point for distinguishing pCR from non-pCR) and the area under the curve was 0.775

The complete regression, TRG0–1 and downstaging occurred in 19 (17.1%), 50 (45.0%) and 42 (37.8%) patients, respectively. The EMD according to nonyCR (7.8 ± 3.2 mm) was significantly higher than yCR (6.1 ± 1.8 mm) ( *p* = 0.033). According to good regression (TRG 0–1 vs. TRG 2–3) and downstaging (ypStage 0-I vs. ypStage II–III), although good regression and downstaging patients showed relatively low EMD, the difference was not significant (* p* > 0.05) (Table 2). Receiver operating characteristic curve analysis revealed a good value for the EMD to predict nonpCR and the area under the curve was 0.775 (Fig 1). For EMD <5.6 mm, the sensitivity for predicting pCR was 75%, with a specificity of 73% and a positive predictive value of 72%.

**Table 2:** EMD according to pCR classification, TRG and downstaging

	patients	EMD	p*
pCR			
pCR	19 (17.1)	6.1 ± 1.8	0.033
non-pCR	92 (82.9)	7.8 ± 3.2	
TRG			
TRG 0–1	50 (45.0)	7.2 ± 3.1	0.355
TRG 2–3	61 (55.0)	7.7 ± 3.1	
Downstaging			
ypStage 0–I	42 (37.8)	7.1 ± 3.0	0.258
ypStage II–III	69 (62.2)	7.8 ± 3.1	

Abbreviations: EMD = extramural depth of tumor invasion; pCR = pathological complete response;

TRG = tumor regression grade.

Data presented as number of patients (n = 111), with percentages in parentheses.

* Determined by Kruskal-Wallis test.

### Correlation between EMD and pathologic tumor response

**Figure 2:**
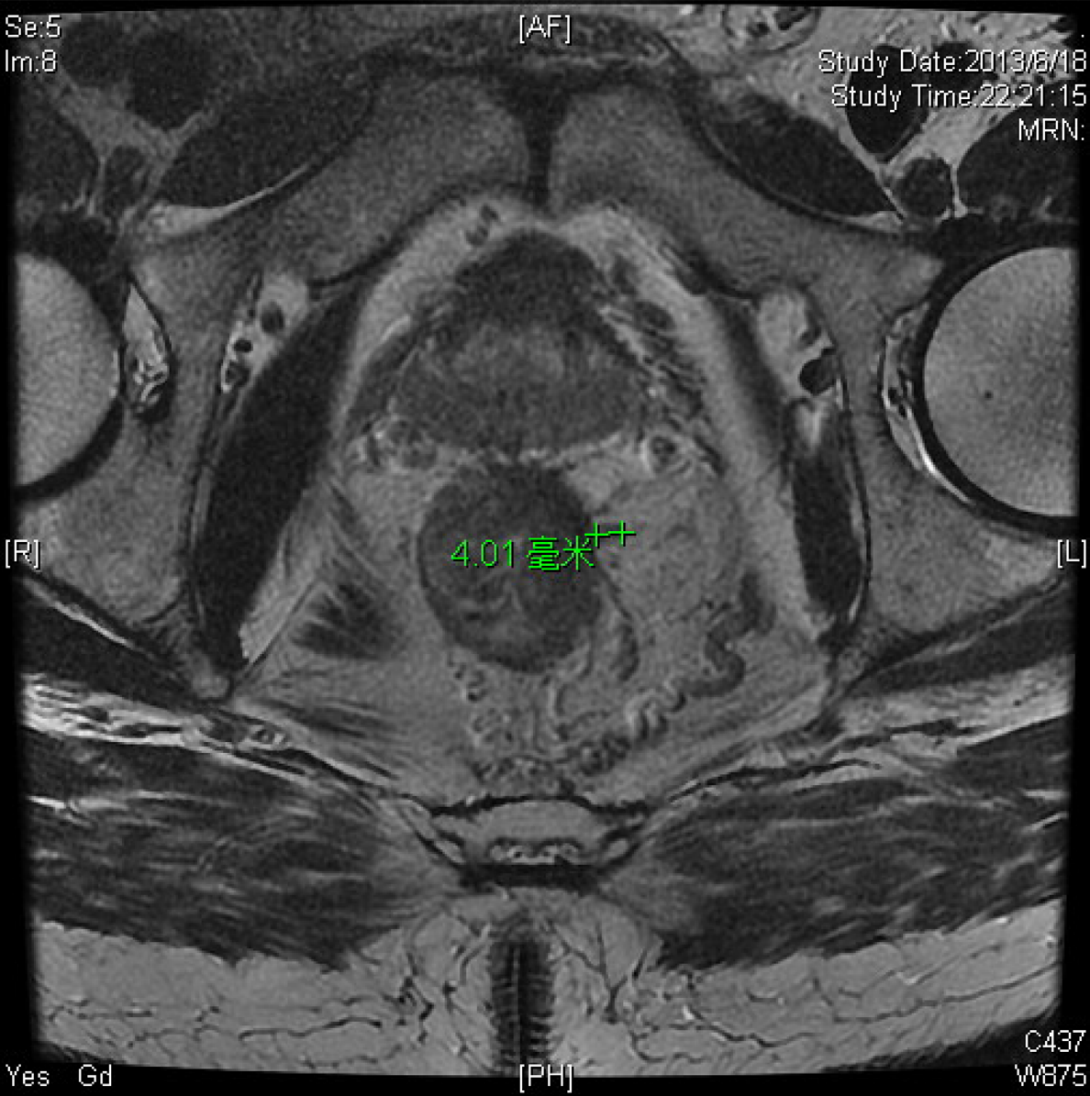

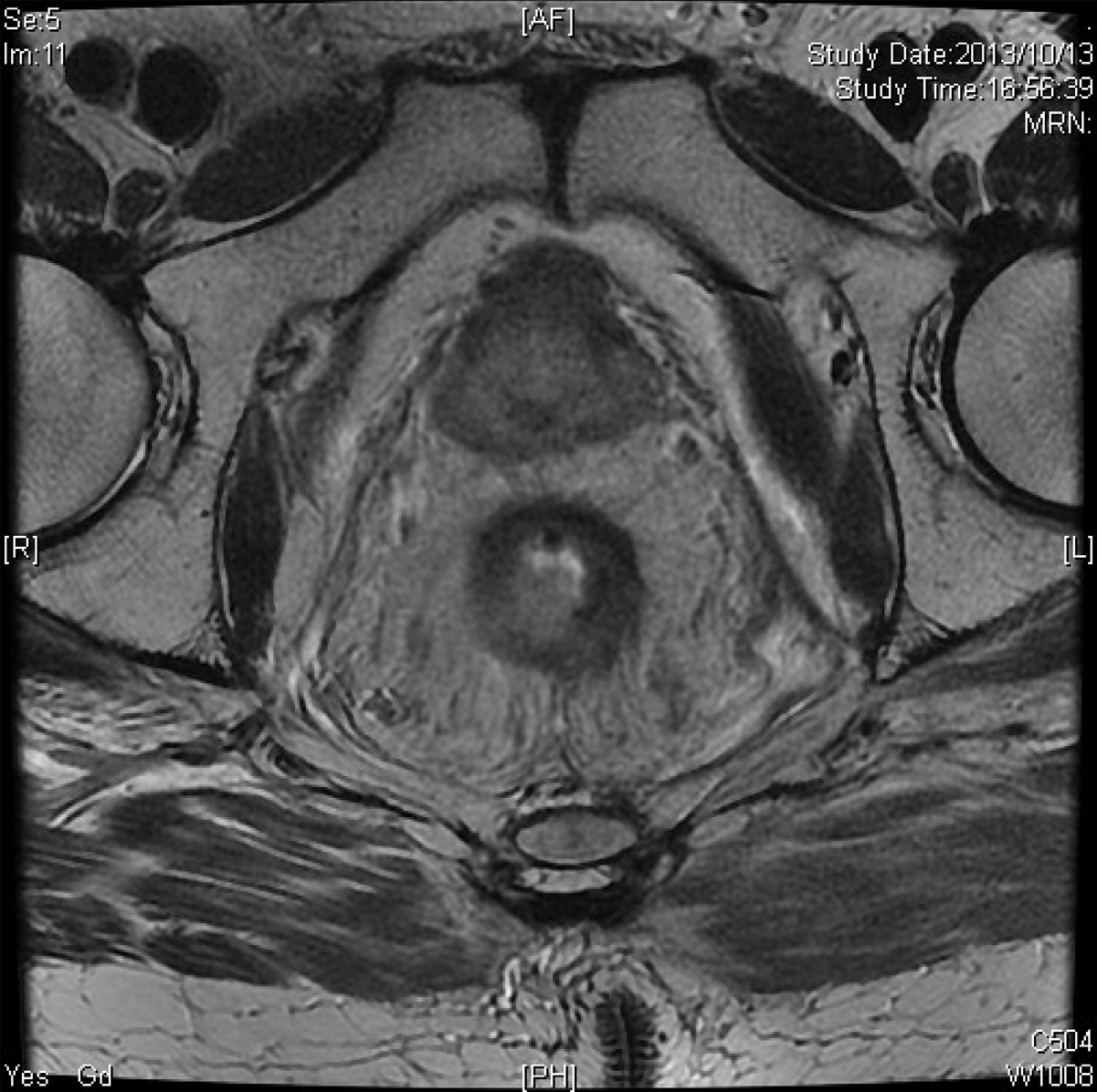
Sixty-three-year-old man with cT3aN0 rectal adenocarcinoma 5 cm from the anal verge Baseline thin section T2-weighted pelvic MRI(a) before CRT revealed posterior rectal lesion. The EMD was 4.0 mm. **b.** axial thin section T2-WI after CRT. Pathological examination of the resection specimen revealed no residual tumor = pCR (pathological complete response) and TRG 0 (tumor regression grade).

**Figure 3:**
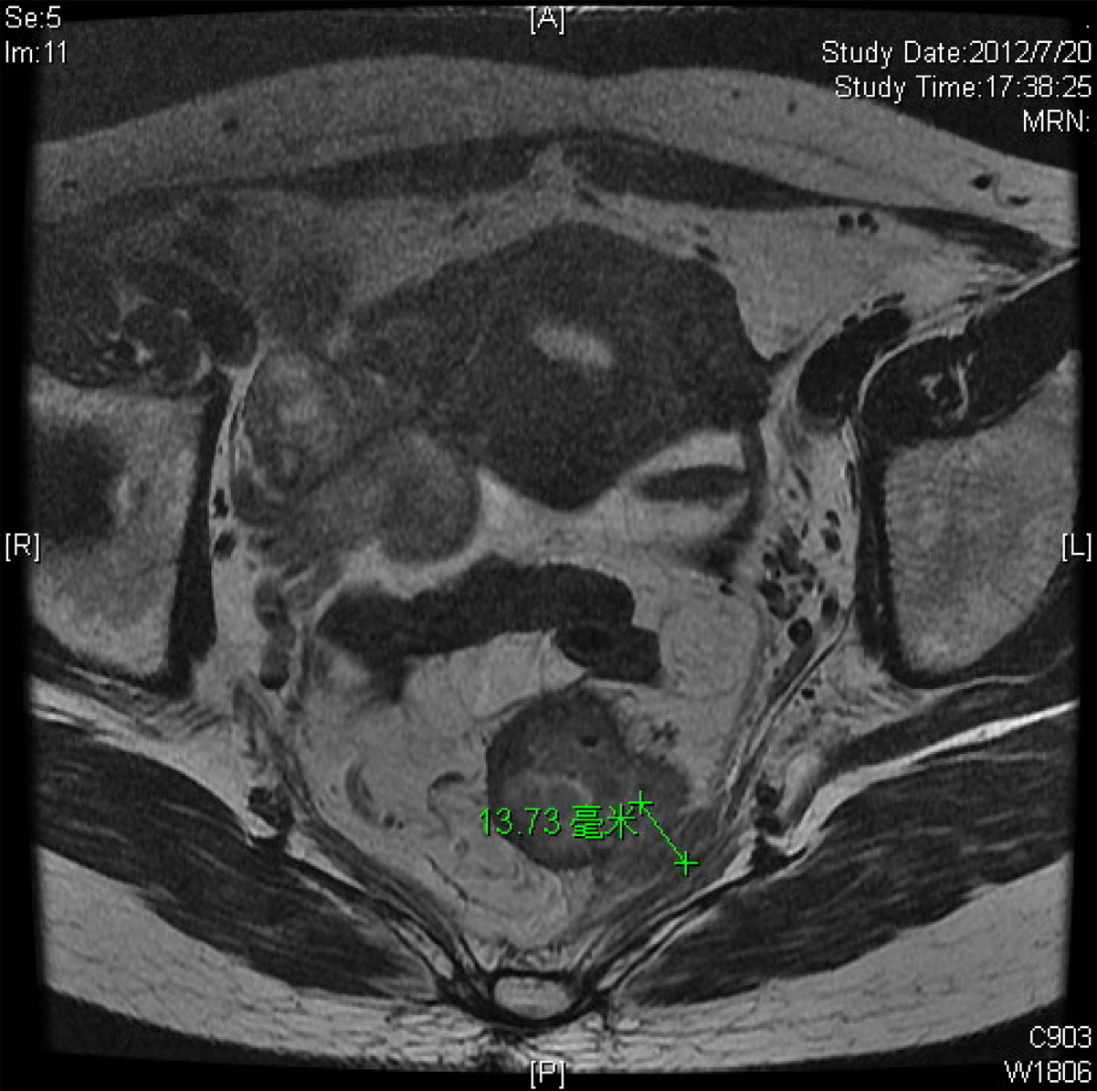

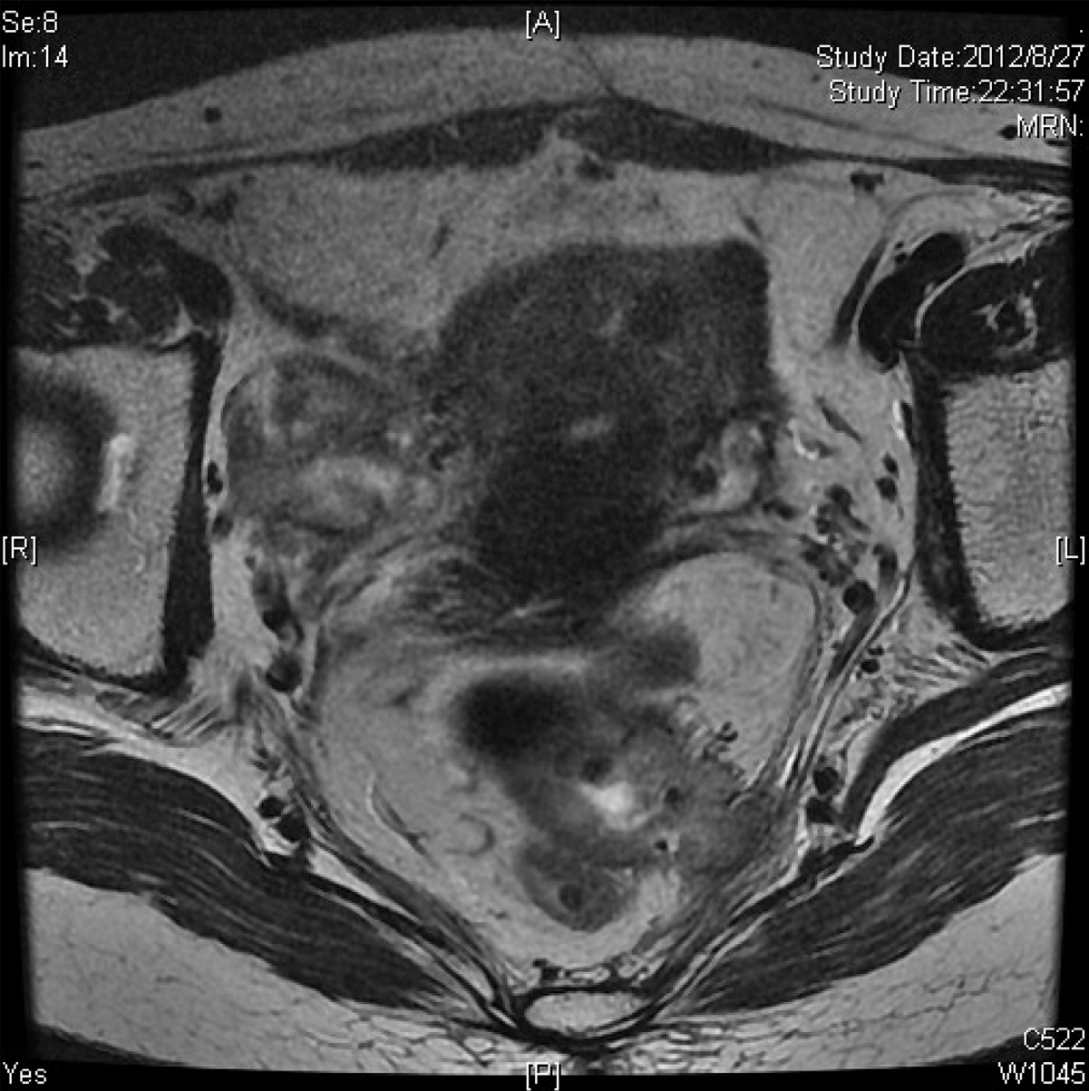
Forty-one-year-old woman with cT3cN0 rectal adenocarcinoma 6 cm from the anal verge Baseline thin section T2-weighted pelvic MRI(a) before CRT revealed circumferential wall thickening. The EMD was 13.7 mm. **b.** axial thin section T2-WI after CRT. Pathological examination of the resection specimen revealed pT3N0 and TRG 3.

The patients were separated into there group according to RSNA's radiology reporting template for primary rectal cancer. The numbers of patients with a EMD <5 mm, 5–10 mm, and >10 mm, were 14 (12.6%), 79 (71.2%), and 18 (16.2%), respectively. The observed rates of pCR, good regression (TRG 0–1, and downstaging (ypStage 0-I) as a function of EMD were listed in Table 3. On univariate analysis, pretreatment EMD was associated with pCR. Compared with patients with a EMD ≥ 5 mm, significantly more patients with EMD <5 mm showed pCR( *p* = 0.019) (Fig 2, 3), while there was no correlation between EMD and good regression or downstaging.

**Table 3:** Observed rates of ypCR, TRG, and downstaging as function of EMD

EMD	Patients		OR	95%CI	P*
pCR	pCR	nonpCR			
<5 mm	5	9	1		
5–10 mm	13	66	0.355	0.102–1.231	0.019
>10 mm	1	17	0.106	0.011–1.050	0.047
TRG	good regression (0–1)	poor regression (2–3)			
<5 mm	9	5	1		
5–10 mm	34	45	0.420	0.129–1.367	0.127
>10 mm	7	11	0.354	0.083–1.502	0.179
Downstaging	ypStage 0–I	ypStage II–III			
<5 mm	8	6	1		
5–10 mm	29	50	0.580	0.185–1.819	0.479
>10 mm	5	13	0.385	0.088–1.673	0.212

Abbreviations: OR = odds ratio; CI = confidence interval; other abbreviations as in Table 2.

* Univariate logistic regression analysis.

## DISCUSSION

In this study we analyzed the EMD of 111 patients with cT3 rectal carcinoma and found significant differences among subgroups with pCR and non-pCR. We measured actual values and found the cutoff point of 5.6 mm to predict pCR. The patients were regrouped into three subgroups according to the degree of EMD T3a <5 mm, T3b 5–10 mm, and T3c >10 mm. The frequency of pCR was significantly greater for patients EMD <5 mm compared with EMD ≥ 5 mm group.

The main limitation of T staging is that T3 tumors comprise the majority of rectal cancers seen at presentation, and the outcome of patients with these tumors depends on the depth of extramural spread. From existing pathologic studies [17–19], it is clear that patients with more than 5 mm of extramural spread should be identified because they have a markedly worse prognosis than do patients who have T3 tumors with 5 mm or less of spread. The prognostic significance of the EMD in rectal cancer was advocated in several articles [1, 20–23], while there were few articles using EMD to predict tumor response to neoadjuvant chemoradiation therapy. In this study, we have demonstrated on pretreatment high-resolution MRI that greater depth of penetration into the mesorectum was independently associated with poor tumor response. The findings on pretreatment MRI were therefore able to stratify patients as good or poor risk for response to neoadjuvant chemoradiation therapy. This permits targeting of good- or poor risk patients for appropriate novel treatment strategies. Recently, Chang GJ et al [24] analyzed 62 pretreatment rectal MRI to determine the MRI assessment of tumor depth was associated to tumor response to neoadjuvant chemoradiotherapy. Their analysis was designed not with the goal of definitively predict pCR, but rather with the aim to classify patients as highly or not likely to exhibit a good response to neoadjuvant therapy before treatment.

Patients with complete treatment response have been considered for organ-preserving nonsurgical treatment strategies [25]. An accurate staging system to predict patients with good-risk tumors is crucial because these groups enable clinicians to provide a tailored adjuvant therapy to patients. There has also been interest in identifying patients for consideration of intensified treatment strategies to improve resectability of patients with poor-risk tumors [26, 27]. In addition, poor treatment response appears to indicate more aggressive tumor biology with poorer long-term outcomes than for patients with a good response; and therefore poor responders may benefit from intensified treatment strategies as well [28–30]. The ability to risk-stratify patients for such treatments depends on an ability to identify them before neoadjuvant treatment initiation. However, our rate of pathologic complete response was 17.1%, consistent with previous reports [27, 28]. A total of 92 (82.9%) of the study patients were observed to have non-pCR to neoadjuvant therapy, the composite criteria of EMD ≥ 5 mm as defined in this study correctly identified 83 (90.2%). ROC curve analysis revealed a good value for the area under the curve and the cutoff of 5.6 mm for EMD could predict pCR. Thus, the pretreatment imaging can be used to identify poor-risk (likely to have non-pCR) patients for novel treatment-intensified protocols. The selection of poor-risk patients is important, which limits the exposure to treatment-related toxicity for the good-risk groups.

The present study had some limitations. First, we only analyzed the correlation between EMD and tumor response by univariate analysis and not designed to predict tumor response with other prognostic factors such as lymph node metastases, angiolymphatic invasion, perineural invasion, and preoperative CEA level by multivariate analysis. Second, the agreement on EMD is only good, even lower than on N stage so there was the potential to overestimate and underestimate tumor depth unless meticulous care was taken to ensure that the imaging plane was orthogonal to the rectal wall and that the images were subsequently carefully interpreted. Third, it would have been clinically interesting to assess the aggressiveness profile of tumors by means of outcome parameters such as disease-free or overall survival. However, this would require a larger patient cohort and a longer follow-up period which was beyond the scope of our current study.

In conclusion, EMD obtained before neoadjuvant chemoradiation is strongly associated with neoadjuvant treatment response. The EMD <5.6 mm is associated with complete pathological response in patients with T3 rectal cancer. This factor should therefore be considered for stratification of patients for novel treatment strategies reliant on pathologic response to treatment or for the selection of good-risk patients for intensified treatment regimens.

## MATERIALS AND METHODS

### Patients

A consecutive cohort of patients with MRI-staged locally advanced (cT3 and cN0–2) rectal cancer treated with preoperative chemoradiotherapy followed by total mesorectal excision surgery at Fudan University Shanghai Cancer Center between March 2010 and December 2013 was identified from the colorectal cancer database, and their records were retrospectively reviewed. All patients had histologically confirmed rectal carcinoma and clinical stage was evaluated according to the 7th AJCC classification. All patients were evaluated before neoadjuvant therapy by physical examination, including digital rectal examination, and flexible endoscopy; computed tomographic (CT) scans of the chest, abdomen; and magnetic resonance imaging of the pelvis. Patients were excluded if they were diagnosed with a non–skin cancer within 5 years of the diagnosis of rectal cancer, did not complete neoadjuvant chemoradiation, did not undergo radical rectal resection, or if the interval from the completion of radiation to surgery was more than 16 weeks. The study received approval from the local institutional ethical committee. The final study population consisted of the 111 patients (79 male, 32 female). Median age was 56 years (range, 23–75 years).

### Treatment

All patients received neoadjuvant concurrent CRT. Radiotherapy (RT) was delivered with a linear accelerator using 6-and 15-MV photons and a three-field technique (posterior-anterior and right and left laterals). Every patient underwent a planning computed tomography (CT) scan in the treatment position (prone position) using a belly board. Three-dimensional conformal RT was used for all patients based on the planning CT, with a total dose of 45 Gy at 1.8 Gy per fraction per day, Monday-Friday. Neoadjuvant chemotherapy was delivered concurrently with RT. Starting on day 1 of RT, patients received capecitabine 625 mg/m^2^ orally, bid (Monday-Friday), and oxaliplatin 50 mg/m^2^ weekly for five consecutive weeks. Surgery was scheduled eight weeks after the completion of CRT. Total mesorectal excision (TME) was mandatory, whereas the form of surgery (anterior resection or abdominal-perineal resection) and whether a temporary colostomy should be performed were decided by the surgeon.

### MR imaging and evaluation

The primary staging MRI was performed before CRT. Patients were imaged in a 3.0 Tesla (T) MR magnet (Signa Horizon, GE Medical Systems, Milwaukee, WI), using a phased-array body coil. The standard imaging protocol consisted of sagittal T2-weighted (T2W) fast spin echo and oblique axial thin-section T2W, which were used for measuring maximal EMD (repetition time/echo time [TR/TE]:3420/110 ms; flip angle: 90o; echo train length: 16; FOV: 20cm; section thickness: 4 mm; number of slices: 20; acquisition time: 6 min 25s). All axial sequences were angled perpendicular to the tumor axis as identified on sagittal MRI which was done by technologists under the guidance of gastrointestinal radiologist. Patients did not receive bowel preparation, antispasmodic medication, or rectal distention before the MR examinations.

Two gastrointestinal radiologists, who were blinded to information obtained at surgery and pathologic analysis, reviewed the T2-weighted imaging set. One professor had more than 10 years and the less experienced professor had 5 years of clinical experience in interpreting rectal MR imaging studies. Each radiologist used a workstation to interpret images and identify the image that depicted the maximal extramural tumor spread. For each tumor, the maximal extramural depth of spread, from the outer edge of the low-signal-intensity longitudinal muscularis propria to the outermost edge of the tumor, was measured and recorded by using the workstation calipers. The final value of EMD was decided by using the mean of the 2 measurements. The cN stage (cN −, N+) was retrieved from MRI at primary staging according to not only the size criteria(>3 mm) but also the irregular margins, T2 or enhancement heterogeneity. The relationship to the mesorectal fascial envelope (circumferential margin, CRM) was also evaluated by them. A measured distance of 1 mm or less on thin-section T2-weighted images was indicative of CRM involvement.

### Histopathologic evaluation

After surgery, the pathologic tumor stage was determined according to the TNM classification system recommended by the International Union against Cancer and the American Joint Committee on Cancer, 7th edition, 2010. Downstaging was determined by comparing the pretreatment clinical and postoperative pathologic classifications and was defined as ypStage 0-I (ypT0–2N0M0; the “yp” prefix indicates final staging after CRT [y] and postoperative pathologic examination [p]). Complete response was defined as the absence of viable adenocarcinoma cells in the surgical specimen (ypT0N0). Tumor regression was graded as follows: Grade 0, no regression; Grade 0, Complete response: No remaining viable cancer cells; Grade 1, Moderate response: Only small clusters or single cancer cells remaining; Grade 2, Minimal response: Residual cancer remaining, but with predominant fibrosis; and Grade 3, Poor response: Minimal or no tumor kill, extensive residual cancer [15]. Regression grading involved both the primary tumor and regional lymph nodes.

### Statistical analysis

Patient, tumor, and MRI characteristics were evaluated with the use of descriptive statistics. The Kruskal-Wallis test was used to assess differences between means of the following groups: pCR versus non-pCR, good regression (TRG0–1) versus poor regression (TRG2–3), downstaging versus non-downstaging. Logistic regression analyses were performed to examine univariate association of subdivided T3 category with pCR, good regression and downstage. To evaluate interobserver agreement regarding the correct measure of tumor EMD value, N stage and CRM evaluation, *k* statistics were used. A *k* value of less than 0.20 indicated poor agreement; a *k* value of 0.21–0.40, fair agreement; a *k* value of 0.41–0.60, moderate agreement; a *k* value of 0.61–0.80, good agreement; and a *k* value of more than 0.81, excellent agreement [16]. For EMD, receiver-operating curves (ROC) was constructed to further investigate the predictive value of EMD and was used to determine a threshold value at which patents with pCR could be distinguished from patients with non-pCR. Statistical analyses were performed using the Statistical Package for the Social Sciences (SPSS, version 21.0).

For all the above mentioned analyses, a *P* value of less than 0.05 was considered statistically significant.
